# Protein P7 of the Cystovirus φ6 Is Located at the Three-Fold Axis of the Unexpanded Procapsid

**DOI:** 10.1371/journal.pone.0047489

**Published:** 2012-10-15

**Authors:** Garrett Katz, Hui Wei, Alexandra Alimova, Al Katz, David Gene Morgan, Paul Gottlieb

**Affiliations:** 1 Sophie Davis School of Biomedical Education, City College of New York, New York, New York, United States of America; 2 Department of Physics, City College of New York, New York, New York, United States of America; 3 Chemistry Department, Indiana University, Bloomington, Indiana, United States of America; University of Pennsylvania School of Veterinary Medicine, United States of America

## Abstract

The objective of this study was to determine the location of protein P7, the RNA packaging factor, in the procapsid of the φ6 cystovirus. A comparison of cryo-electron microscopy high-resolution single particle reconstructions of the φ6 complete unexpanded procapsid, the protein P2-minus procapsid (P2 is the RNA directed RNA-polymerase), and the P7-minus procapsid, show that prior to RNA packaging the P7 protein is located near the three-fold axis of symmetry. Difference maps highlight the precise position of P7 and demonstrate that in P7-minus particles the P2 proteins are less localized with reduced densities at the three-fold axes. We propose that P7 performs the mechanical function of stabilizing P2 on the inner protein P1 shell which ensures that entering viral single-stranded RNA is replicated.

## Introduction

The cystoviridae family of viruses, of which φ6 was the first discovered, contain three segments of double stranded RNA. The RNA packaging, replication, transcription mechanism, and overall structure resembles that of the reoviruses making the species an excellent model system for these important pathogens.

**Figure 1:**
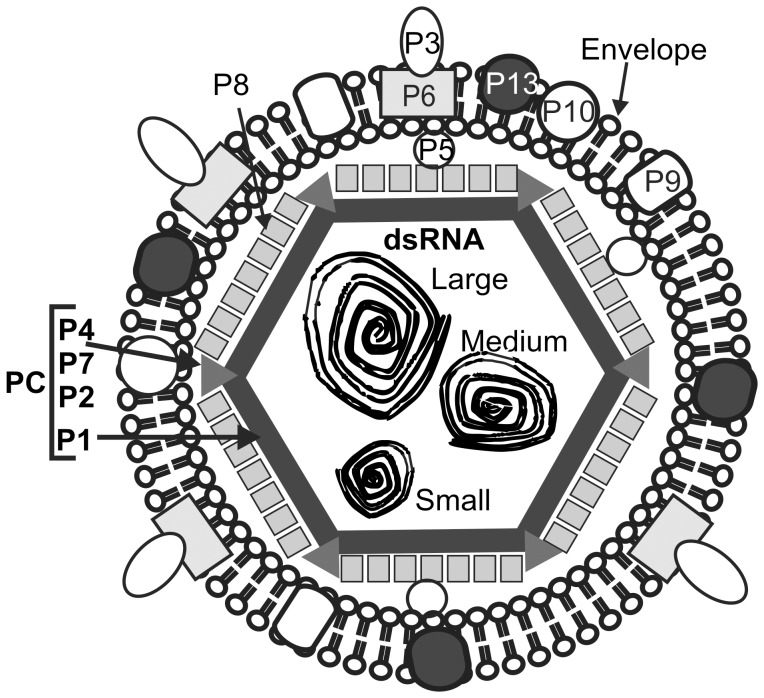
Schematic diagram of φ6. Schematic diagram of φ6 showing major elements of the virus.

The initial step in cystoviridae replication is the assembly of a closed and unexpanded procapsid (PC). The PC is responsible for RNA packaging, transcription, and genome replication and is composed of four proteins - P1, P2, P4, and P7 [1], [2]. A schematic of φ6 is shown in Fig. 1, the hexagon in the center, less RNA, represents the PC. The PC is initially assembled as a dodecahedron with recessed vertices prior to RNA packaging [3]. The 120 copies of P1 form the procapsid shell [4]. During replication, the PC first forms in the unexpanded state, followed by sequential expansion as ssRNA is packaged. Isolated mutants of expanded or unexpanded PC particles can be produced in *Escherichia coli* [2]. Recently, tomographic reconstructions of cryo-electron micrographs from both unexpanded and expanded PCs showed that all vertices in a individual PC are either in a completely unexpanded or completely expanded state [5]. During RNA packaging, the expansion proceeds in a stepwise mechanism that accommodates the outward pressure of packaged RNA [5]. The packaging of the cystovirus genomic ssRNA into a preformed PC depends on the three portal proteins, P2, P4 and P7. The portals are located at the 12 PC five-fold vertices. These proteins must function in a coordinated mechanism to package the viral RNA segments, replicate them into a double-stranded form and transcribe them [2]. P2 is the RNA-dependent RNA polymerase (RdRP). P4 is the hexameric nucleotide triphosphorylase (NTPase) packaging motor [6]. P4 is assembled on the outer PC surface at the 12 potential RNA portal sites. The six-fold symmetry of P4 forms a mismatch with the five-fold axis of symmetry [7], [8]. P7 is assembled into the PC as well, and is required for efficient PC assembly [9], RNA packaging [10], [11] and transcription [9].

**Figure 2:**
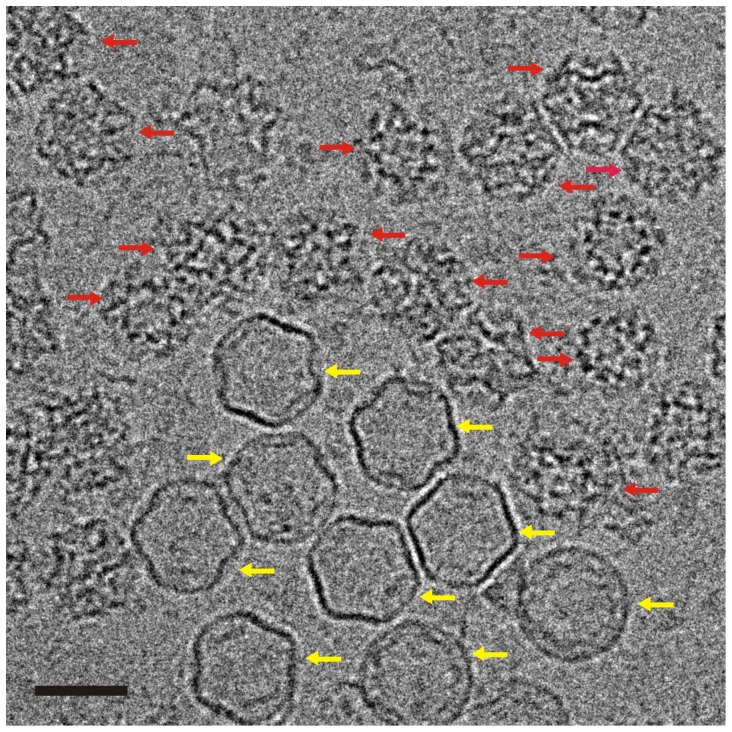
Projection image of complete PC. Typical projection image showing expanded (yellow arrows) and unexpanded (red arrows) P1247 particles. Scale bar is 50 nm.

P7 is the least characterized of the PC proteins. In solution, there is evidence that it forms an elongated dimer [9], [12], [13]. Poranen *et al*. observed that an excess concentration of P7 accelerated assembly of P1 *in vitro*, indicating that P7 may stabilize P1 [13]. The precise position of P7 within the PC, and hence a structural explanation for its importance has not been described [14]. Cryo-EM studies of related cystovirus, φ12, indicate that after expansion, P7 surrounds the P4 hexamer on the five-fold axis of symmetry [15]. The crystal structure of the φ12 P7 core-proteins was obtained at 1.8 Å resolution by x-ray diffraction [16] and together with solution nuclear magnetic resonance (NMR) studies suggest a variety of functional roles for P7. P7 exists as a unique α/β-fold and forms a symmetric homodimer in solution. The C-terminal tail (amino acids 129–169) is significantly disordered but on interaction with RNA shows a reduced degree of disorder. The NMR measurements show that the flexible C-terminal tail minimally interacts with the protein core. Of great interest in that work was the suggestion that P7 could play a role in viral RNA recognition. Chemical shifts in the NMR spectrum of the P7 C-terminus (amino acids 159 to 163) were evident in the presence of 5-nucleotide oligoribonucleotides corresponding in sequence to the 5′-ends of plus-sense φ6 mRNA. The most significant chemical shifts were the negatively charged amino acids Glu135, Glu143, and Asp160. This observation suggests that P7 mechanistically interacts with P2 during RNA packaging and replication. The location of P2 in the expanded PC remains controversial but based upon reconstructions of empty and filled φ12 PC, it is suggested that it is directly beneath the central position of the five-fold symmetry axes [15]. Cryo-EM studies have shown that the RNA polymerase of reoviruses are attached to the core shell and overlap the five-fold axis [17]. In the unexpanded φ6 PC, P2 is located on the three-fold axis between the inverted five-fold vertices [18]. These studies suggest that P2 in the unpackaged PC is initially at the three-fold axis but during RNA packaging and replication, translates to a position beneath the five-fold symmetry axis. Recent cryo-EM studies by Nemecek *et al.* [19] suggest that the P2 and P4 sites are randomly incorporated into the PC and no mechanism exists to cause these two proteins to co-localize. Proteins homologous to P7 could exist in reoviruses where, for example, μ2 serves as an RNA polymerase cofactor [20].

In this paper we utilized cryo-electron microscopy with single particle reconstruction to locate the position of protein P7 near the three-fold symmetry axis within the unexpanded, empty PC. Difference mapping techniques using reconstructions of P2 and P7 negative particles identify the position of P7. The results suggest that P7 may stabilize the P2 RdRP in its position at the three-fold axis in empty-unexpanded φ6 PC. These observations are consistent with, and explain, the functions attributed to P7 - in particular the stabilization of unexpanded PCs, regulation of RNA replication, and the movement to the five-fold vertices in filled PCs (“motion model”). Our findings have significant implications in regard to both the expansion of the PC and efficient RNA replication during genomic packaging in segmented dsRNA viruses.

**Table 1:** Particle Count and Resolution.

	Particles Count	Resolution	Defocus Range
P1247	1778	15 Å	1.28–2.82
P124	1802	16 Å	1.23–3.04
P147	1762	15 Å	1.13–3.66

## Materials and Methods

### Preparation of Procapsids

Procapsids were produced in transformed *Escherichia coli* using the plasmids pLM687, pLM574, and pLM1906 to co-express the proteins that assemble procapsids [2], [21]. Following the naming convention of Sen *et al.* [18], the populations are designated: P1247– complete PC containing P1, P2, P4 and P7; P147– PC lacking the P2 polymerase; and P124– PC lacking P7. Overnight cultures of plasmid-transformed cells were grown in LB medium for 2–3 hours with 0.5 mM isopropyl β-D-thiogalactopyranoside and lysed with a French Press. The procapsid particles were purified by zone gradients (10 to 30% sucrose) as described by Gottlieb *et al.* [11]. Sucrose was removed from the PC samples by dialysis in Buffer A [11]. Buffer A contains 10 mM KH_2_PO_4_ (pH 7.5) and 1 mM MgSO_4_. It was noted that for all three populations, large aggregates were observed which contained both expanded and unexpanded particles. It was determined that maintaining the buffer at pH 7.5 in 150 mM NaCl throughout the entire isolation procedure increased the fraction of unexpanded particles.

**Figure 3:**
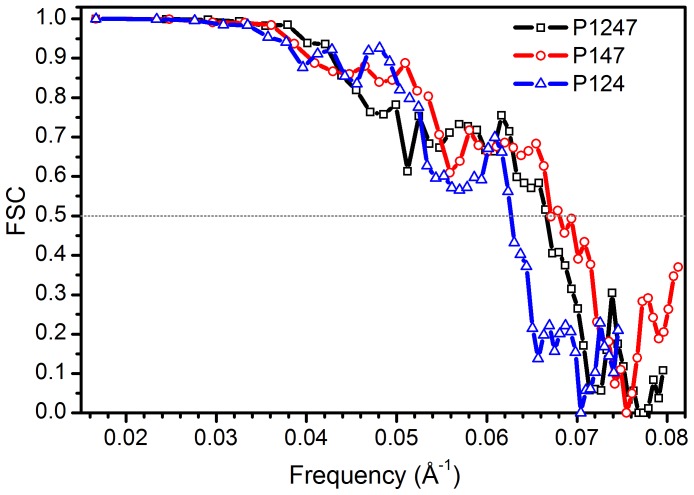
FSC curves. FSC for P1247, P124 and P147 showing resolution of the reconstructions.

### Cryo-electron Microscopy

Samples for electron microscopy were plunge frozen in liquid ethane on copper grids with holey carbon film, and imaged with a JEOL 2100 microscope operating at 200 kV. Images were taken at 1.5, 2.0, and 2.5 nominal underfocus and 48000 magnification for all three PC populations. Images were recorded on Kodak electron image film and scanned with a Zeiss SCAI scanner at 14 µm per pixel resolution. At 48000 magnification, each pixel represents 2.9 Å.

**Figure 4:**
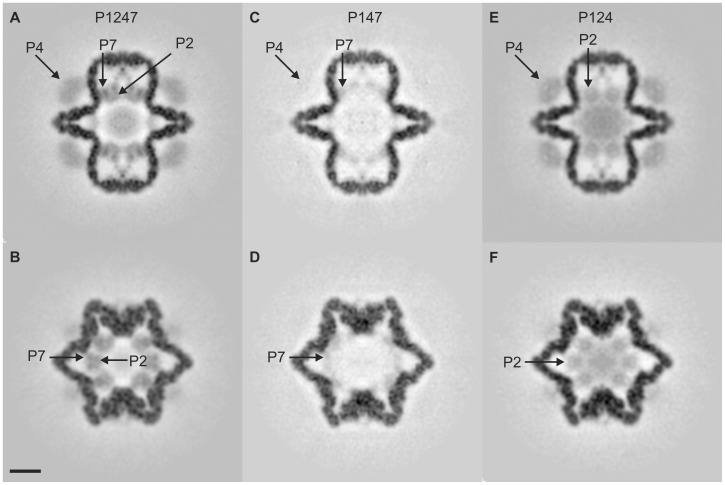
P1247, P147 and P124 reconstruction slices. Slices through the P1247, P147, and P124 reconstructions viewed along the 2-fold icosahedral symmetry axis. A, C and D are central slices; B, D and F are offset 43 Å. Arrows indicate positions for one of the P2, P4 and P7 proteins. Scale bar is 10 nm.

### Single Particle Reconstruction

Only isolated, unexpanded particles were included in the reconstructions. A typical projection image is shown in Fig. 2 containing both expanded and unexpanded PCs. Scanned micrographs were converted to MRC format and particle coordinates were selected in IMOD [22]. Averaged power spectra were generated for each micrograph using SPIDER [23] and inspected visually to screen for astigmatism and drift. Deficient micrographs were excluded from further processing. CTF parameters were calculated using CTFFIND3 [24]. Boxed particles were converted to PIF format and preprocessed for normalization, linear gradient and blemish removal, using the RobEM program in the AUTO3DEM package [25]. Initial models were created using the random model method and refined with AUTO3DEM [25]. Resolutions for each map were determined by Fourier Shell Correlation (FSC).

**Figure 5:**
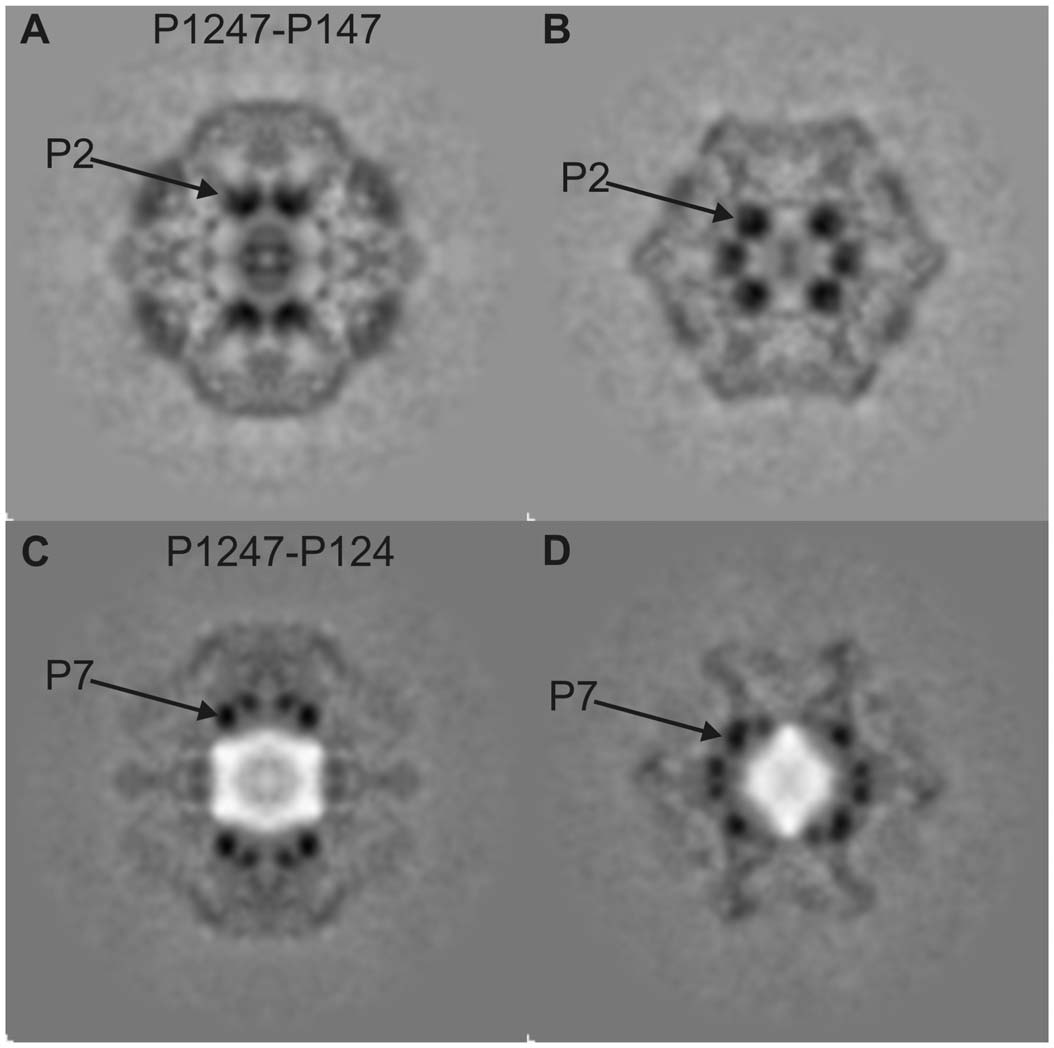
Difference Maps. Difference maps produced by subtracting P147 (A, B) and P124 (C, D) from the P1247. A and C are central slices, B and D are offset 43 Å. Arrows point to one of the P2 and one of the P7 in the respective panels.

### Difference Maps

Comparable numbers of P1247, P124 and P147 particles (Table 1) were used in each reconstruction so as to provide approximately equivalent resolution and signal-to-noise ratios that were utilized in the generation of the difference maps. To suppress artifacts due to differences in resolution, each reconstruction was low-pass filtered in XMIPP [26] to a resolution of 1.6 nm, which was the lowest resolution of the three reconstructions as determined by FSC. Density scaling was performed assuming the background and P1 shell densities were equal for the three reconstructed particles. The P1 shell was identified by determining the threshold for each reconstruction so that the enclosed volume matched the expected volume of P1, estimated based on a molecular mass of 84 kDa, protein density of 1.41 g/cm^3^ [27], and copy number of 120. The background was identified by a spherical annulus excluding both the particle and the apodization zone at the boundary of the reconstructions. The three reconstructions were all scaled to have a mean value of 0 and equal standard deviations within the union of the background and the P1 region. RobEM was then used to determine radial scale factors, based on the radial averages of the P1 masks. However, it was found that there was no significant change in the resulting difference maps for radial scale factors based on the P1 shell or the complete reconstructions.

**Figure 6:**
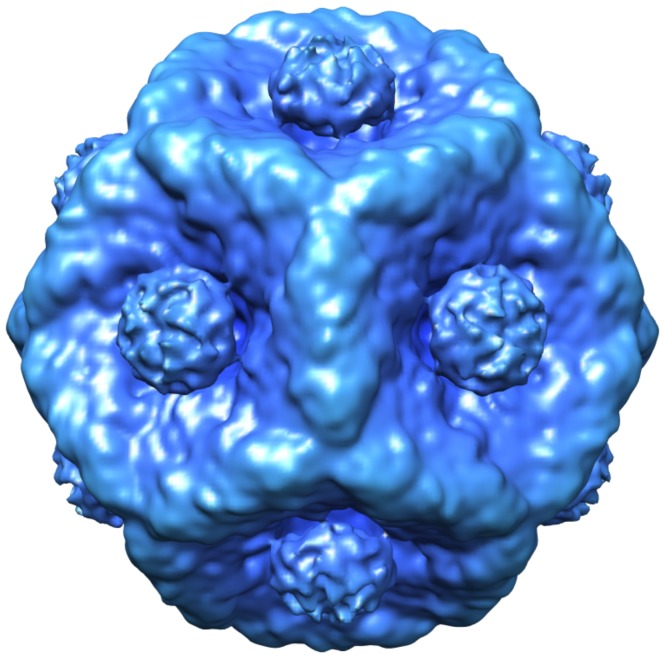
Isosurface rendering. Isosurface rendering of unexpanded P1247.

**Figure 7:**
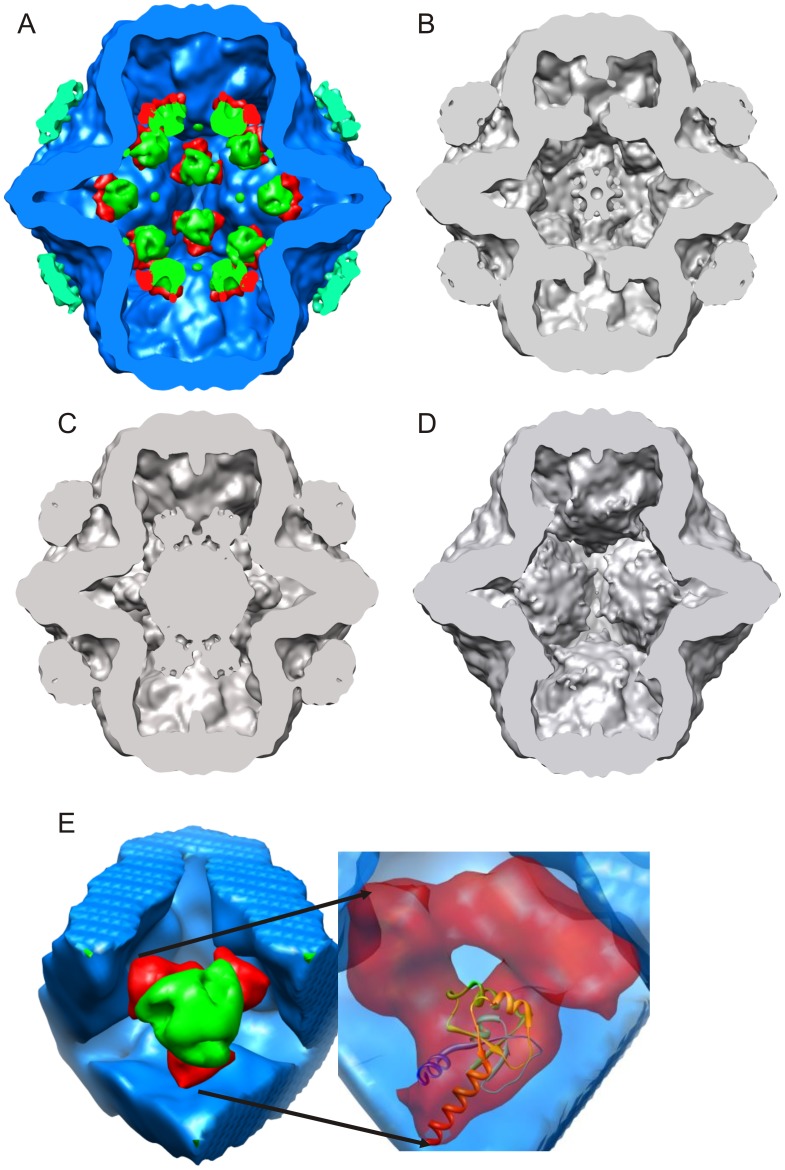
Central view of isosurfaces maps. A) Isosurface rendering of P1247 based on difference map segmentation of the PC into P1 (blue), P2 (green), P4 (blue-green), and P7 (red), protein locations can be determined from the rendering. B) Isosurface rendering of P1247. C) Isosurface rendering of P124. D) Isosurface rendering of P147. E) Magnified section showing P2 surrounded by three P7 monomers and an insert showing docked atomic model of P7. All particles are unexpanded.

The scaled maps for P124 and P147 were each subtracted from P1247 to yield the corresponding difference maps. A P14 map was generated by subtracting P1247 from the sum of the P124 and P147 maps. These locations were separated manually by use of a spherical mask for illustrative purposes in the superimposed difference maps. All isosurfaces were rendered with USFC Chimera [28], using a threshold of 2 standard deviations for the superimposed difference maps and 1 standard deviation for P1247. If the superimposed difference maps are all summed, the result is equivalent to the original P1247 reconstruction: (P1247– P124) + (P1247– P147) + (P124+ P147– P1247) = P1247. The difference maps are not summed but contoured individually to produce their respective isosurfaces. For this reason, different thresholds are required for a comparable visualization.

### P2 and P7 Occupancy

The occupancy of P2 and P7 was estimated from their respective densities using the highest density region of the P1 shell as 100% occupancy and the background as 0% occupancy. P2 and P7 densities were determined by generating masks from the difference maps and averaging the densities in the mask volume. This technique only counts occupancy in the nominal P2 and P7 positions and does not account for shifted positions in the P124 or P147 mutants.

## Results and Discussion

### 

#### Single Particle reconstructions of unexpanded procapsids

The unexpanded and expanded PCs can be readily classified by visual inspection (Fig. 2) and only non-overlapping, unexpanded particles were included in the reconstructions. The fraction of unexpanded particles was 93% for P1247, 70% for P147 and 91% for P124 in the preparations.

Three-dimensional density maps were created by single particle reconstructions of P1247, P124 and P147. Particle numbers and resolutions of the reconstructions are summarized in Table 1 using an FSC cutoff of 0.5 (Fig. 3). The size of the P7 monomer was estimated by simulating a density map from the P7 protein data bank file. The longest dimension is ∼4.8 nm and the shortest dimension ∼2.8 nm, therefore the achieved resolution of the reconstructions (1.6 nm) is sufficient to identify and locate P7 densities.

Figs. 4A–F shows cross-sections of the reconstructed volumes of P1247, P124, and P147, at two views which show significant features – a central slice (Figs. 4A, C, E), and a slice offset 43 Å (Figs. 4B, D, F) from the center. The complete reconstructions are in the Supplementary Material (Video S1-P1247.avi, Video S2-P124.avi and Video S3-P147.avi). The P1247 map (Fig. 4A, B) shows all the structural elements of the procapsid. We compare the P2-minus and P7-minus particles to the complete P1247 particle in order to determine locations of P2 and P7.

A comparison of P147 densities to P1247 provides insight into the position of the cofactor P7, as well as the level of structural interdependency between P7 and the polymerase P2. P147 reveals a residual density near the three-fold axes indicative of P7 as shown in Figs. 4C, D. P7 appears to be in contact with the P1 inner shell and may project into it. The densities corresponding to P7 are relatively weak partially due to its substoichiometric occupancy. P7 disorder may also contribute to its reduced density. The lost densities at the three-fold axes in the P147 particles, confirm that P2 normally occupy these locations, consistent with the work of Sen *et al.* [18] and Nemecek *et al.* [19]. In addition to the three-fold axes lost densities, P147 exhibits a reduced density at the particle center which suggests that the diffuse central density of P1247 procapsid is largely composed of P2. The “central” P2 density is less evident at a 43 Å offset in P1247, supporting the notion that the diffuse P2 is found in the central region. Surprisingly, it is noted that the P4 density is significantly reduced in P147. To our knowledge, there is no structural link between P2 and P4. It has previously been reported that P1-only and P1–P7 particles are very unstable and difficult to isolate [29], therefore it is likely that the lack of P2 results in a less stable particle in which P4 occupancy is diminished during assembly.

The distribution of P2 inside the P1 shell can be determined by an inspection of the P124 density map (Figs. 4E, F). The densities at the three-fold axes, taken in conjunction with the corresponding P7 densities (Fig. 4C, D) indicate that the complete particle densities at the three-fold axes are complexes of the P2 polymerase and the P7 cofactor. The P124 particle exhibits an enhanced, more diffused, central density, indicating a more disordered state for P2 than in the complete PC. The P2 densities at the three-fold axes appear to be less than in the P1247 particle. Additionally, the disordered P2 locations in the P7-minus particles suggest that P7 holds the P2 RdRP at the three-fold axes of an unpackaged and unexpanded PC. It is observed that P124 does not exhibit a reduced density for P4 while when P2 is completely missing, P4 densities are reduced. The explanation for this is not clear since P14 particles can be isolated [29].

### Difference Maps

Difference maps were generated to produce an initial segmentation of the entire PC into its constituent proteins and enumerate the potential protein sites. The difference maps highlight the features discussed above. Figs. 5A, B show the P1247-P147 difference map for central and 43 Å offset slices, respectively. Due to reduced P4 occupancy in P147, the resulting difference map (P1247– P147) indicates P2 as well as P4 densities. The complete 3-dimensional difference maps are in the Supplementary Material (Video S4-P1247 P124 Difference Map.avi, the P1247 minus P124 difference map; and Video S5-P1247 P147 Difference Map.avi, the P1247 minus P147 difference map). P2 locations are identified as high density regions near the 20 three-fold axes. A count of P2 densities in the complete 3-D difference map indicates 20 locations in the unexpanded PC. However, based on studies of the related φ12 virus, the expanded PC contains only 12 potential P2 sites [15], [30], therefore, at most 12 of the 20 unexpanded P2 sites can be occupied and thus the maximum P2 occupancy in the unexpanded PC is 0.6.

The P1247-P124 difference maps for the central and 43 Å offset slices are shown in Figs. 5C, D, respectively. The high density regions corresponding to P7 are readily identified in each map. The difference map has significant regions of negative density (i.e. regions in which the P124 densities are greater than the P1247 densities). The negative densities are most likely a consequence of random locations of P2 in P124 that are unoccupied in the P1247 indicating that P2 may have a higher concentration in the central region of the P124 particle than in the complete PC. This may be a consequence of higher P2 production in the P124 recombinant particle.

### Segmentation of the Isosurface Rendering

In isosurface rendering of the entire P1247 is presented in Fig. 6. In order to visualize the P2 and P7 locations, it is first necessary to mark the P1 and P4 densities. A difference map containing only P1 and P4 (not shown) is generated by subtracting the map of P1247 from the sum of the P124 and P147 maps (P124+ P147– P1247 = P14). Due to the low P4 density in P147, the P14 map is primarily P1. P4 was segmented manually from the P2 difference map (P1247– P147) using a spherical mask. Isosurface renderings for the P1 and P4 map, the P2 map (P1247 minus P147), and the P7 map (P1247 minus P124) are superimposed, producing a segmentation of the PC with its constituent proteins identified. Figs. 7A, B show central slice isosurfaces for the superposition map and the P1247 map, respectively. The complete isosurface map is in the Supplementary Material (Video S6-Isosurface.avi). The sum of the superposed difference maps is mathematically identical to P1247, but by contouring them separately, one has the advantage of identifying specific locations for the individual proteins. In Fig. 7A, P7 is coded red, P1 is coded blue, and P2, located inside the shell, is coded green. P4 densities, located at the five-fold axes (2, 4, 8 and 10 o’clock in Fig. 7A) are color coded blue – green. The P4 densities are to some extent reduced in the P2 difference map due to the lower P4 occupancy in the P147 particles. Figs. 7C, D show the equivalent isosurface for P124 and P147, respectively. The central high density region of P124 represents disordered P2 which does share this location in P1247. This density is reflected as the negative in the difference map shown in Figs. 5C and D. The complete absence of P2 densities is observed in the P147 rendering.

Fig. 7E shows a close-up view of one of the P2/P7 complexes at a three-fold axis. The complexes consist of three P7 surrounding each P2. P7 appears to form a bracket holding P2 to the P1 inner shell. This co-localization may provide a mechanism for P7 to maintain P2 in a pre-RNA packaged position relative to the inner P1 shell. An atomic model of the monomeric P7 core structure (PDB entry 2q82) [16] is shown in a ribbon rendering, positioned inside the isosurface rendering of the cryo-EM density (Fig. 7E insert). Chimera was used to fit the φ12 P7 crystal structure to the EM density map and the correlation coefficient was 0.75 for the position shown in the image. The ordered P7 X-ray structure (amino acids 129 to 169) is seen to plausibly fit in the density from the EM reconstruction. The P7 dimer that is seen in solution may not be the structure of the protein when it is assembled in the PC as indicated by the docking fit in our density difference map.

### P2 and P7 Occupancy

From a comparison of density maps, the P2 occupancy in the complete P1247 is found to be 50% - close to the unexpanded PC maximum of 12 P2s. In the P124 particle, the P2 occupancy is 28%. This reduced P2 occupancy is more likely a result of shifts in P2 location from its nominal site rather than less P2 in the P124 mutant. In P1247, the P7 occupancy is 57% but is only 19% in the P147 mutant, indicating that in the absence of P2, there is less P7. Although these occupancy measurements are relatively crude, they are consistent with our expectation for P2 in the complete PC, and the relative changes in P2-minus and P7-minus PC are quite striking.

### Conclusion

Our placement of P7 near the three-fold axes agrees with the multiple function model attributed to the protein [14]. P7 appears to perform the mechanical function of “holding” P2 in position near the three-fold axis. This “holding” function could make P2 most accessible to the packaging mRNA that is replicated to dsRNA. The observation that P2 appears less ordered in P124 reconstructions (Figs. 4E, F and 5A, B) further argues that P7 provides a bracket for P2. We propose that P7 interacts with P2 to form a lock which inhibits P1 shell movement prior to RNA packaging which might stabilize an unexpanded PC. This model supports the hypotheses put forth by Butcher *et al.* [3] and Eyrilmaz *et al.* [15], [16] that P7 is part of a hinge for P1 expansion during RNA packaging. We acknowledge that since P2 and P7 occupancy is less than 100%, in a given particle P2 and P7 may occupy different three-fold sites. However, the P2 disorder in the P7-minus particle and the reduced P7 occupancy in the P2-minus particle indicate that P2 and P7 may be coupled and maintain each other in place. Therefore, it is likely that P7 is not a static component of the PC. It is mobile and might play a role in grappling the RNA polymerase (P2) during PC assembly and maintaining the polymerase position near the three-fold axis prior to RNA packaging and replication. Given the probability (from NMR measurements) of an interaction of the P7 C-terminus with the 5'-ends of the plus-sense φ6 mRNA, it is not unreasonable to consider an additional RNA packaging role in which P7 guides viral RNA to the P2 polymerase [16].

Our result that P7 is attached to the P1 shell (Figs. 4C, D and 5C, D) indicates that during packaging and replication of the dsRNA, P2 could decouple from P7 in the expanded PC. This is consistent with our prior observations on φ12 [15] that P2 translates from the three-fold to the five-fold axes after dsRNA packaging and replication. This model also predicts a lowered expansion threshold for P2-minus and P7-minus particles due to a less constrained vertex brace.

## Supporting Information

Video S1 **Tomogram of P1247, the complete procapsid.**
(AVI)

Video S2 **Tomogram of P124, P7-minus particle.**
(AVI)

Video S3 **Tomogram of P147, P2-minus particle.**
(AVI)

Video S4 **Difference Map of P1247 minus P124.**
(AVI)

Video S5 **Difference Map of P1247 minus P147.**
(AVI)

Video S6 **3-D Isosurface rendering of P1247 showing locations of the four PC proteins.**
(AVI)
